# Cardiac Telerehabilitation After Heart Attack Using E-Learning Platforms and Monitoring Cardiovascular Risk Factors: A Narrative Review of the Literature

**DOI:** 10.3390/medicina61040635

**Published:** 2025-03-30

**Authors:** Dragoș Trache, Liviu Ionuț Șerbănoiu, Mircea Ioan Alexandru Bistriceanu, Gabriel Olteanu, Octavian Andronic, Liviu Călin, Ștefan-Sebastian Busnatu

**Affiliations:** 1 Bagdasar-Arseni Clinical Emergency Hospital, 041915 Bucharest, Romania; dragos-alin.trache@rez.umfcd.ro (D.T.); liviu-ionut.serbanoiu@drd.umfcd.ro (L.I.Ș.); liviu-stefan.calin@drd.umfcd.ro (L.C.); stefan.busnatu@umfcd.ro (Ș.-S.B.); 2 Department of Cardio-Thoracic Pathology, Faculty of Medicine, “Carol Davila” University of Medicine and Pharmacy, 050474 Bucharest, Romania; 3 “Carol Davila” University of Medicine and Pharmacy, 050474 Bucharest, Romania; 4 Innovation and eHealth Center, “Carol Davila University of Medicine and Pharmacy” Bucharest, Strada Pitar Moș 20, 030167 Bucharest, Romania; octavian.andronic@umfcd.ro

**Keywords:** telerehabilitation, myocardial infarction, cardiovascular risk factors, cardiac rehabilitation, telemedicine

## Abstract

This narrative review aims to evaluate the current evidence on the use of cardiac telerehabilitation (CTR) in patients after myocardial infarction, focusing on the effectiveness of e-learning platforms and remote monitoring for addressing cardiovascular risk factors, improving physical fitness, and enhancing patient adherence. The review also explores the limitations and gaps in the literature, highlighting the need for future research to optimize CTR approaches. A comprehensive literature search was conducted using PubMed and Scopus, focusing on specific keywords. The search yielded fifteen randomized controlled trials. Data from these studies were analyzed to evaluate the methodology, interventions, patient characteristics, and outcomes related to the use of CTR in managing cardiovascular risk factors and improving physical fitness. The included studies demonstrated that CTR interventions, delivered via online platforms, phone calls, and smart devices, were effective in improving cardiovascular risk factors, physical activity levels, and overall patient satisfaction. CTR appears to be associated with improvements in exercise tolerance, VO_2_ max, body composition, and adherence. While the outcomes were promising, there is still limited evidence regarding the long-term impact of CTR on cardiovascular risk factors and lifestyle interventions, particularly in non-exercise components like dietary management and psychological support. Cardiac telerehabilitation presents a feasible and effective alternative to traditional in-hospital rehabilitation programs for patients recovering from myocardial infarction. The integration of e-learning platforms and smart devices enhances patient adherence, improves cardiovascular risk factors, and increases access to rehabilitation services, particularly for those who face barriers to traditional care. However, further large-scale studies are needed to establish standardized protocols and best practices for CTR. Additionally, future research should address disparities in access to digital health technologies, especially among rural and underserved populations, to ensure equitable access to these innovative approaches.

## 1. Introduction

Cardiovascular diseases (CVD) continue to be the leading cause of morbidity and mortality worldwide, despite the significant prevention strategies and therapeutic advances in modern medicine. Following a cardiac event, especially post-ST-elevation myocardial infarction (STEMI), non-ST-elevation myocardial infarction (non-STEMI), or after percutaneous coronary intervention (PCI), clinical guidelines recommend a thorough evaluation of the patient’s clinical status to determine their eligibility for enrollment in supervised cardiac rehabilitation programs (CR) [1,2]. These guidelines strongly support CR as a Class 1, level of evidence A recommendation by the American Heart Association/American College of Cardiology (AHA/ACC) and European Society of Cardiology (ESC), indicating the highest level of evidence for its efficacy in reducing mortality and improving outcomes [3,4,5,6]. However, despite these firm recommendations, CR remains underutilized [7,8]. There is low adherence among patients, compounded by insufficient rehabilitation centers to meet the overwhelming demand of cardiovascular patients, highlighting a critical gap between guideline recommendations and real-world practice. Addressing this gap is essential for improving patient outcomes post-cardiac events.

To enhance accessibility and improve patient adherence to CR programs, innovative approaches such as online home-based cardiovascular telerehabilitation programs have been developed [9]. These programs allow patients to engage in rehabilitation remotely, under clinical supervision, using digital platforms. Additionally, hybrid CR models have emerged, combining traditional in-hospital or specialized center-based sessions with online rehabilitation sessions [10,11].

In recent years, mobile health (mHealth) and telemedicine have emerged as essential tools in both primary and secondary cardiovascular prevention, providing innovative solutions to improve patient outcomes and healthcare efficiency. Telemedicine encompasses a broad spectrum of digital health interventions, including mHealth, telemonitoring, and electronic health (eHealth), each contributing to personalized and accessible cardiovascular care [12]. In primary prevention, mHealth interventions enhance lifestyle modifications, improve adherence to cardiovascular risk reduction, and boost exercise capacity, as demonstrated by their impact on peak VO_2_ levels in high-risk patients [13]. In secondary prevention, telemedicine supports CR, chronic disease management, and early detection of cardiovascular events, helping to reduce hospital readmissions and improve outcomes [12].

Telerehabilitation involves the delivery of rehabilitation services via telecommunications and internet networks, providing a critical framework for patients in subacute or chronic stages of rehabilitation. It helps reduce hospitalization times and associated costs by enabling access to comprehensive rehabilitation programs, which can serve as alternatives to traditional methods. The telerehabilitation process mirrors the traditional structure of rehabilitation programs: patient assessment, goal setting, and the design of a personalized rehabilitation plan, involving close interdisciplinary collaboration among various healthcare professionals [14].

Cardiovascular telerehabilitation (CTR) represents a modern and accessible strategy for managing cardiovascular diseases. This innovative, multidisciplinary approach offers an effective alternative to traditional rehabilitation programs, which are typically conducted in specialized centers or hospitals. Studies have shown that CTR achieves comparable improvements in functional capacity, VO_2_ max, and cardiovascular risk reduction to traditional center-based programs [15,16,17]. Moreover, it improves adherence through flexible, remote monitoring, reducing logistical barriers while remaining a cost-effective alternative to in-hospital rehabilitation [17,18].

Beyond its application in CTR, telemedicine has demonstrated significant potential across various cardiology domains, enhancing access to specialized care and improving patient outcomes. In heart failure management, remote monitoring technologies such as implantable hemodynamic sensors and telemonitoring programs facilitate early detection of decompensation, allowing timely intervention that reduces hospitalization and mortality rates [19,20]. Similarly, the feasibility and effectiveness of virtual visits (VVs) for cardiac electrophysiology patients were demonstrated during the COVID-19 pandemic, showing comparable clinical outcomes to in-person visits while improving accessibility and patient satisfaction. VVs reduced the need for in-office evaluations, allowed real-time symptom assessment, and maintained patient engagement, suggesting that a hybrid model of care combining virtual and in-person visits may represent an optimal approach in post-pandemic healthcare [21].

**Figure 1:**
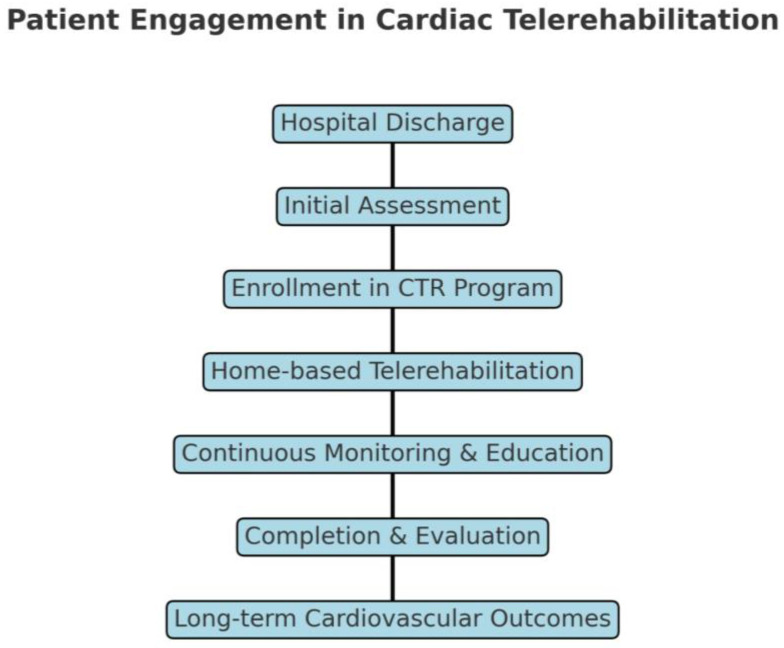
Patient engagement in CTR. The flowchart maps the dynamic patient journey through CTR: from the moment of hospital discharge and progressing through initial assessment, program enrollment, home-based rehabilitation, continuous monitoring, completion and evaluation, and ultimately long-term cardiovascular outcomes.

To provide a clear and concise overview of how patients progress through CTR, we have developed a visual flowchart illustrating the standard engagement pathway (Figure 1).

Although these technologies show promise in primary prevention settings, there remains a significant gap in the research regarding their application within the framework of CR, especially in addressing a holistic range of interventions, including exercise, diet, psychological support, and risk factor modification. This disparity suggests that while digital tools are effective in risk management for prevention, more evidence is needed to establish their efficacy in a rehabilitation context where recovery (rehabilitation), rather than prevention, is the main focus.

This narrative review aims to evaluate the current evidence supporting the use of CTR for managing post-myocardial infarction patients, focusing on addressing not only exercise-based interventions but also essential components such as cardiovascular risk factor control, nutritional counseling, psychosocial management, patient education, and vocational counseling. The review seeks to highlight the gaps in the literature regarding the effectiveness of CTR in these non-exercise domains and explore its potential for improving access and outcomes through the use of digital health technologies. By examining both the strengths and limitations of current research, this review aims to provide a comprehensive understanding of the role of CTR in holistic cardiovascular care.

## 2. Materials and Methods

We conducted a comprehensive search of the electronic databases PubMed and Scopus on September 22, employing the following keyword combinations: “Cardiac telerehabilitation” OR “Telemedicine” AND “Myocardial infarction” OR “Heart attack” OR “Acute coronary syndromes” AND “Home” AND “E-learning platforms” AND “Cardiovascular risk factors.” This search yielded only two randomized controlled trials (RCTs), which were subsequently included in our analysis.

A subsequent search was conducted using an alternative keyword combination: (“Cardiac telerehabilitation” OR “Telemedicine” OR “Telerehabilitation”) AND (“Myocardial infarction” OR “Heart attack” OR “Acute coronary syndromes”). This search yielded 221 articles without applying specific filters for article type, but restricting the publication period to 2018–2024 and limiting the language to English. After reviewing the titles and abstracts, 53 publications were identified as relevant to our research focus. These 53 articles were further analyzed, during which inclusion and exclusion criteria were applied for subsequent in-depth analysis.

The study selection and data extraction process was conducted independently by two reviewers (D.T. and L.I.S.). Any disagreements were resolved through discussion, and if consensus was not reached, a third reviewer (L.C.) was consulted to make a final decision.

To ensure a comprehensive analysis, data were collected on the methodology and research protocol, including the number of participants, sex, age, smart devices used, inclusion and exclusion criteria for participants, communication channels with the CR team, and recommendations regarding physical activity and lifestyle interventions (e.g., dietary guidelines and stress management strategies). Additionally, the outcomes reported by the investigators were documented, with particular attention given to the efficiency, feasibility, and safety of CTR interventions in modifying cardiovascular risk factors (CVRF) (Table 1). Thus, we aimed to evaluate the clinical outcomes and the practical aspects of implementing CTR, including participant adherence and the overall impact on health behavior modifications.

Following the final reanalysis, our review includes fifteen studies that align with our subject, along with three additional studies (a retrospective study, a cross-sectional study, and one observational study) that support the need for implementing cardiac telerehabilitation as a screening tool.

## 3. Results and Discussion

Fifteen studies were included in this narrative review and analyzed based on predefined objectives [22,23,24,25,26,27,28,29,30,31,32,33,34,35,36], complemented by three additional studies: a retrospective study [37], a cross-sectional study [38], and an observational study [39], which underscore the importance of integrating smart devices as screening tools.

While the included studies collectively suggest the potential of cardiac telerehabilitation (CTR), a deeper examination reveals notable heterogeneity in methodologies, intervention formats, and outcomes, which necessitates a critical evaluation of their findings. The majority of studies employed existing platforms or simple communication tools like phone calls and messaging services for lifestyle interventions, physical training, and periodic assessments.

In contrast, two studies developed specialized applications for real-time patient monitoring and delivering comprehensive cardiac rehabilitation (CR) components. Notably, Li et al. [22] utilized a 5G Internet of Things (IoT)-enabled smart device system integrated with the Healthy Life Cycle application. The intervention demonstrated significant improvements in cardiovascular risk factors (CVRF), including metabolic equivalents (METs), VO_2_ max, HDL-C, and BMI, alongside reductions in anxiety and depression scores. Importantly, compliance rates (80.8% in the intervention group) reflected the efficacy of personalized, technology-driven approaches. However, limitations in Li et al.’s study—notably its small sample size and short duration—raise concerns about the robustness of these findings. While improvements in intermediate markers like VO_2_ max and HDL-C are promising, the lack of long-term follow-up data diminishes confidence in the sustained impact of these interventions. Additionally, the absence of significant changes in LDL cholesterol underscores a gap in addressing all aspects of lipid management, highlighting the need for more comprehensive and sustained interventions.

In contrast, Krzowski et al. [29] employed a mobile application targeting post-AMI patients but observed limited outcomes, including no significant differences in hospital readmissions or emergency care utilization. While reductions in smoking rates and trends toward improved blood pressure values were noted, these changes lacked statistical significance. The findings suggest that stand-alone mobile applications may not adequately address systemic or behavioral barriers, underlining the importance of integrating human interaction or additional support mechanisms. Similarly, Zullig et al. [32] compared nurse-administered telephone interventions with web-based approaches for blood pressure management. While modest reductions in systolic blood pressure (SBP) were observed, only nurse-led interactions achieved statistically significant improvements. This highlights the significant role of personalized human engagement in promoting adherence and achieving meaningful health outcomes, which digital tools alone may not provide.

Several studies highlighted the benefits of using wearable devices and online platforms to monitor physical activity and CVRF, but the results varied widely. For instance, Mitropoulos et al. [26] and Calvo-López et al. [23] both demonstrated significant improvements in VO_2_ max, physical activity duration, and adherence rates using telehealth platforms. However, their findings are constrained by small sample sizes, short durations, and single-center designs, limiting their generalizability. While Calvo-López et al. emphasized improvements in adherence to Mediterranean dietary patterns and reductions in anxiety, depressive symptoms showed no significant changes, suggesting that the psychological impact of CTR warrants further exploration.

The randomized trial by Treskes et al. [31] demonstrated high patient satisfaction and engagement with CTR, with 96% of participants valuing access to personal health data. This underscores the importance of patient autonomy in driving adherence. However, the study’s focus on low-risk patients and lack of long-term follow-up data limit its applicability to broader populations, particularly those with complex comorbidities.

Digital interventions also hold significant promise in addressing mental health challenges such as depression and anxiety, which are prevalent among post-myocardial infarction patients. Recent studies have demonstrated that digital mental health tools are moderately to highly effective in reducing depression and anxiety symptoms in low- and middle-income countries, suggesting their potential applicability in broader settings [40]. The bidirectional relationship between mental health and cardiovascular outcomes underscores the importance of integrating psychological support into CR programs. Particularly, internet-based cognitive behavioral therapy (iCBT) effectively reduces symptoms of depression and anxiety in cardiac patients. A randomized controlled trial involving post-myocardial infarction patients with depressive or anxiety symptoms found that a 14-week iCBT program significantly improved mental health outcomes compared to usual care [41]. Additionally, a systematic review highlighted that digital health interventions (DHIs) could offer viable solutions to treat depression and anxiety in patients with heart disease, although further research is needed to establish their long-term efficacy [42].

Hence, addressing mental health alongside CR may improve patient adherence to rehabilitation protocols and overall well-being. Future research should explore the long-term benefits of these interventions, particularly their impact on reducing hospital readmission rates and improving patient-reported quality of life (QoL). Additionally, further studies should assess patient engagement and adherence to digital interventions to optimize their effectiveness and scalability in routine clinical practice [43].

Further reinforcing the role of DHIs in secondary prevention, a recent meta-analysis encompassing 13 studies and 7657 patients provided compelling evidence of their clinical benefits. The analysis demonstrated that DHIs significantly reduce all-cause mortality and hospital readmission rates while enhancing adherence to cardiovascular medications, thus, underscoring their potential to improve long term outcomes [44]. This evidence is consistent with the expanding body of research advocating for the integration of mHealth and telemedicine into routine cardiovascular care.

### 3.1. Methodological Variability

The reviewed studies exhibit significant methodological variability, complicating direct comparisons and data synthesis. Differences in intervention intensity, ranging from structured, supervised programs to self-guided recommendations, make it difficult to assess the precise impact on health outcomes. Exercise modalities also varied, with studies prescribing aerobic, resistance, or multimodal training, and differing in adherence requirements. Additionally, wearable device variability—involving diverse smartwatches, fitness trackers, and biosensors with inconsistent accuracy and data outputs—further limits comparability.

Without clear standards, digital health research risks becoming a patchwork of incomparable findings. Aligning intervention intensity, exercise prescription, and device selection under a unified framework would transform fragmented data into a cohesive body of evidence, driving more reliable conclusions and practical applications.

### 3.2. Long-Term Outcomes and Follow-Up Limitations

Inconsistencies in study methodologies not only affect immediate findings, but also raise concerns about long-term data reliability.

Furthermore, the quality and functionality of smart devices varied, introducing potential biases in data reliability and clinical outcomes. Long-term outcomes, an essential metric for evaluating CTR, remain underexplored. Most studies focused on intermediate markers such as VO_2_ max, HDL-C, and SBP, with limited evidence on major adverse cardiovascular events (MACE) or mortality. This gap underscores the need for longitudinal, multicenter studies to establish the long-term efficacy and safety of CTR.

While current studies demonstrate improvements in intermediate markers, and physical activity levels, the absence of long-term follow-up data limits our ability to assess CTR’s impact on MACE, mortality, and healthcare utilization. Few studies extend beyond six to twelve months, making it difficult to determine whether the observed benefits translate into sustained cardiovascular protection. Furthermore, while some interventions show improvements in patient adherence and engagement, the long-term clinical significance of these behavioral modifications remains unclear.

As was mentioned above, the absence of large-scale, multicenter trials further complicates the generalizability of existing findings. Future research should focus on long-term studies evaluating CTR’s effect on primary endpoints such as MACE, rehospitalization rates, and overall survival. Moreover, integrating standardized outcome measures across trials would facilitate cross-study comparisons and meta-analyses, ensuring a more robust understanding of CTR’s long-term benefits. We believe that by addressing these important gaps, CTR can be positioned not only as a convenient rehabilitation tool, but also as a validated, long-term strategy for cardiovascular risk reduction and secondary prevention.

### 3.3. Equity and Accessibility in CTR

While CTR offers a promising approach to increasing access to rehabilitation services, its implementation is not without challenges. Socioeconomic factors, geographical disparities, and digital literacy play essential roles in shaping participation rates and outcomes [45,46]. Recent evidence suggests that despite the potential of CTR to overcome logistical barriers, it may inadvertently exclude certain patient populations, exacerbating existing healthcare inequities. A prospective analysis of CTR participation by Brouwers et al. (2022) revealed that non-participants were significantly older, less educated, and more likely to have pre-existing CVRFs such as smoking and reduced exercise capacity [47]. Among the most frequently reported barriers to participation were insufficient technical skills or a lack of interest in digital health (26%) and a preference for center-based rehabilitation (21%) [47]. This suggests that DHIs are not always a suitable replacement for in-person care, particularly for individuals with limited familiarity or comfort with technology.

Regarding socioeconomic and geographic disparities, low-income populations and individuals in rural or remote areas face unique barriers to CTR. While these programs aim to reduce the burden of travel and improve accessibility, inadequate internet infrastructure and limited access to smart devices remain significant obstacles. Patients from lower socioeconomic backgrounds often have lower engagement with digital health technologies, which may stem from financial constraints, limited access to reliable internet, and lower digital literacy [45,46,47]. Furthermore, individuals living in remote areas may struggle with inconsistent network coverage, limiting the effectiveness of real-time monitoring and teleconsultations.

Disparities in healthcare extend beyond connectivity issues. Patients with lower educational attainment and those without prior exposure to digital tools are less likely to engage with mHealth applications or wearable devices. Lower educational levels are a strong predictor of non-participation in CTR, emphasizing the urgent need for targeted digital literacy programs and patient-centered design strategies that accommodate diverse user needs [47].

A key limitation in current CTR interventions is the assumption that patients are proficient in using digital health tools. Brouwers et al. highlighted that older patients, in particular, were less likely to engage with CTR due to technological concerns [47]. To address this, CTR programs must incorporate user-friendly interfaces with simplified navigation and step-by-step guidance. Comprehensive onboarding and training programs can help patients build confidence in using telehealth platforms, while hybrid models that integrate in-person and virtual support allow patients to receive assistance when needed.

To ensure CTR is accessible and equitable, healthcare systems must adopt a multifaceted approach that goes beyond simply providing remote rehabilitation services. Leveraging offline-compatible technologies, such as downloadable rehabilitation materials or SMS-based interventions for patients without reliable internet access, could improve accessibility. Integrating CTR with local healthcare facilities or community centers where patients can receive assistance may further support adoption, particularly in underserved populations. Personalized intervention models that consider a patient’s individual needs, preferences, and comfort with digital tools could improve adherence and outcomes. Many patients report missing the peer interactions and group dynamics found in traditional center-based rehabilitation. Developing interactive platforms that incorporate social engagement features, such as peer-to-peer support groups or gamification strategies, may help bridge this gap and improve patient motivation.

Sex and age disparities in CTR adoption remain significant, reflecting a complex interplay of biological, socioeconomic, and systemic barriers. Women, despite experiencing equal or even greater benefits from CR, remain 36% less likely than men to enroll [48]. This disparity is largely driven by lower physician referral rates, greater caregiving responsibilities, and systemic biases within healthcare delivery [49]. Moreover, women face a higher prevalence of multiple comorbidities, which can complicate adherence, and are less likely to receive aggressive secondary prevention strategies despite having comparable cardiovascular risk [50].

Beyond sex-based disparities, older adults also face critical challenges in accessing CTR. Patients over 65 years are significantly less likely to engage in remote rehabilitation programs, largely due to low digital literacy, difficulties in navigating health applications, and skepticism about the effectiveness of remote care [51,52]. These factors disproportionately affect women and minority populations, further exacerbating health inequities.

To bridge these gaps, CTR interventions must integrate age- and sex-sensitive approaches that proactively address barriers to participation. Research highlights the effectiveness of systematic referral mechanisms, which significantly increase CR enrollment rates among women when compared to traditional physician-driven referral processes [48].

To fully harness the transformative potential of CTR in expanding access and mitigating logistical constraints, it is imperative to address the systemic inequities that may inadvertently limit its reach. Without targeted interventions, digital health advancements risk deepening existing healthcare disparities rather than alleviating them. Future research should prioritize the development of inclusive digital literacy initiatives, tailored engagement strategies for underrepresented populations, and hybrid rehabilitation models that seamlessly integrate technological innovation with personalized, patient-centered care.

### 3.4. Technological Challenges and Wearable Devices Standardization in CTR

The precision and reliability of wearable device data remain concerns, with variations in device quality potentially impacting clinical decision-making. Differences in sensor sensitivity, calibration methods, and data transmission protocols can lead to inconsistencies, potentially resulting in inaccurate clinical assessments. Notably, commercially available wearable devices often lack rigorous regulatory oversight, contributing to variability in reported physiological parameters such as heart rate, oxygen saturation, and activity/inactivity levels.

To enhance the reliability of CTR interventions, it is imperative to develop standardized protocols for device validation, ensuring consistency in data collection and interpretation across studies. Recent efforts have been made to guide researchers and clinicians in selecting reliable wearable devices. A practical framework for evaluating continuous monitoring wearable devices has been proposed, incorporating key criteria such as continuous monitoring capability, device suitability, accuracy and precision, feasibility, and cost [53]. This step-by-step selection process aligns with regulatory initiatives like the U.S. Food and Drug Administration (FDA) guidance on digital health technologies for remote data acquisition [54] and the European Medicines Agency (EMA) guidelines on computerized systems and electronic data in clinical trials [55].

Protecting patient data privacy is paramount, especially as digital health platforms collect and process increasingly sensitive medical information. Within the European Union, the General Data Protection Regulation (GDPR) serves as a critical safeguard, enforcing rigorous encryption protocols, explicit patient consent mechanisms, and transparent data-handling practices to uphold the highest standards of security and trust [56].

Globally, the International Medical Device Regulators Forum (IMDRF) provides unified regulatory guidance for medical devices, including software as a medical device (SaMD), a key component of CTR technologies that depend on remote monitoring and real-time feedback [57]. Also, several ISO standards ensure security and quality in digital health. ISO 27001:2013 [58] strengthens cybersecurity in digital rehabilitation platforms, ISO 13485:2016 [59] sets quality management standards for medical devices, and the ISO 82304 [60] series establishes reliability benchmarks for health software, guiding its design, evaluation, and safety [61].

Further efforts are needed to establish universally accepted benchmarks for smart device accuracy and clinical applicability. Regulatory bodies are working toward improving validation protocols, but future studies should focus on integrating artificial intelligence (AI)-driven quality control measures and real-time calibration features to mitigate discrepancies in device-generated data. As digital health continues to redefine CR, the future lies in refining these technologies to bridge the gap between innovation and clinical reliability.

### 3.5. Non-Exercise Components in CTR

It is well established that a comprehensive CR program consists of multiple core components, each of which plays an essential role in improving patient health, facilitating the recovery process and preventing acute exacerbations [62]. Specifically, these core components include individualized exercise prescription based on clinical and biological status of the patient, aerobic and resistance training, nutritional counseling, psychological support, body composition management (including weight loss, muscle mass, visceral and body fat, and hydration levels), and CVRF management (such as hypertension, smoking, alcohol consumption, dyslipidemia, and diabetes) [62]. However, when transitioning to a CTR ecosystem, the focus is predominantly placed on physical activity, often neglecting other essential components such as dietary and psychological interventions.

Despite the proven benefits of CTR, most studies primarily focus on exercise-based interventions, with limited emphasis on dietary counseling, psychological support, and other non-exercise components [26,63]. While some studies have assessed adherence to heart-healthy diets, such as the Mediterranean diet, their integration into structured CTR programs remains inconsistent [23]. Digital platforms have the potential to incorporate nutrition counseling through mobile applications, teleconsultations with dietitians, and AI-driven personalized meal planning, yet research evaluating the long-term effectiveness of such interventions remains scarce. Similarly, psychological support, particularly in the form of iCBT, has shown promise in managing post-myocardial infarction anxiety and depression [41]. However, the lack of standardized protocols for mental health integration in CTR suggests that future studies should explore its scalability and long-term efficacy.

A key reason for the underrepresentation of these non-exercise components in CTR literature is the methodological variability across studies, making it difficult to compare interventions and outcomes [63]. Additionally, research often prioritizes short-term physiological markers such as VO_2_ max and blood pressure changes rather than broader QoL measures, adherence to lifestyle modifications, or mental health improvements. Future studies should aim to incorporate more comprehensive endpoints that account for the multifaceted nature of CR. Expanding CTR beyond exercise-based programs to include structured nutritional and psychological support may enhance patient engagement, improve long-term adherence, and ultimately lead to better cardiovascular outcomes.

### 3.6. Cost-Effectiveness of CTR

While the feasibility of CTR is well established, its economic viability compared to traditional center-based CR remains a critical factor in broadening adoption. Cost-effectiveness analyses suggest that CTR is not merely a convenient alternative but a financially sustainable and scalable model, particularly when considering long-term healthcare expenditures, patient productivity, and societal costs.

Recent economic evaluations underscore the cost-saving potential of CTR while maintaining comparable health outcomes to traditional CR. A cost–utility analysis from the SmartCare-CAD RCT found that although initial intervention costs were slightly higher for CTR due to telemonitoring infrastructure, total healthcare-related expenses were lower over time. The study reported a mean cardiac healthcare cost reduction of 720 EUR per patient in CTR group compared to center-based CR, albeit without statistical significance [64]. Moreover, from a societal perspective, which includes indirect costs like absenteeism and informal caregiving, CTR demonstrated a 3887 EUR lower overall expenditure per patient, reinforcing its cost-effectiveness as a sustainable healthcare model.

Similarly, a systematic review of RCT on exercise-based CTR concluded that telerehabilitation is at least as cost-effective as conventional CR, with multiple studies reporting significant reductions in intervention costs [65]. The review found that across multiple trials, CTR consistently exhibited lower per-patient costs due to decreased facility expenses, optimized resource allocation, and improved patient adherence.

Beyond direct healthcare expenditures, CTR minimizes indirect economic burdens associated with traditional CR. Center-based programs require frequent in-person visits, which translate into substantial travel expenses, time away from work, and also caregiving responsibilities. The SmartCare-CAD study reported a 2553 EUR per patient reduction in unpaid labor costs and absenteeism from work among CTR participants [64]. Batalik et al. found that home-based CTR patients saved significantly on travel expenses, with cost reductions ranging from 82 to 408 EUR per patient, making it a viable option for populations with mobility constraints or limited access to rehabilitation centers [65].

Moreover, CTR appears to reduce unplanned healthcare utilization. Batalik et al. found that CTR participants incurred significantly lower medication costs, with an average of 159 EUR per patient reduction in pharmaceutical expenses [65]. Thus, CTR’s capacity for real-time monitoring and early intervention may help prevent exacerbations, reducing costly hospital admissions and long-term disease progression.

Cost-effectiveness evaluations of CTR often express outcomes using the Incremental Cost-Effectiveness Ratio (ICER), which quantifies the additional cost per Quality-Adjusted Life Year (QALY) gained. The systematic review by Batalik et al. highlighted that CTR was cost-effective in 92% of analyzed studies, with an ICER as low as −21,707 EUR per QALY, indicating that telerehabilitation was dominant (both less costly and more effective) in certain cases [65].

From a Return-on-Investment (ROI) perspective, funding CTR programs could yield significant savings for healthcare systems and insurers. Future research should focus on longitudinal economic evaluations and real-world implementation studies to further validate its fiscal sustainability and optimize reimbursement strategies.

### 3.7. Limitations

Importantly, this narrative review study has several limitations that warrant consideration. First, the heterogeneity in methodologies, including variations in intervention formats, exercise modalities, and the use of digital tools, poses challenges for drawing consistent conclusions and limits the generalizability of findings. Many of the included studies have small sample sizes, which reduce statistical power and the robustness of their outcomes. Furthermore, the lack of standardized protocols across studies introduces variability, particularly in the design and implementation of exercise programs, dietary counseling, and psychological support. Another notable limitation is the short duration of most studies, which prevents an assessment of the long-term impact of cardiac telerehabilitation on cardiovascular risk factors, major adverse cardiovascular events, or mortality. Additionally, disparities in access to technology, particularly among rural and underserved populations, highlight potential inequities that could limit the scalability of these interventions. Variability in the quality and reliability of smart devices further complicates the interpretation of outcomes, as inconsistencies in data accuracy could bias clinical results. Lastly, the limited focus on non-exercise components, such as psychological and dietary interventions, suggests an incomplete exploration of the holistic needs of post-myocardial infarction patients. Addressing these limitations through larger, multicenter studies with standardized protocols and long-term follow-up is essential to strengthen the evidence base for CTR.

### 3.8. Future Research Directions

Maximizing the potential of CTR requires a shift toward more comprehensive research that moves beyond small-scale trials. Large, multicenter studies are necessary to confirm findings and ensure that CTR strategies can be applied across diverse populations and healthcare settings. The absence of standardized protocols for exercise interventions, dietary guidance, and psychological support continues to hinder meaningful comparisons, thus establishing clear guidelines as a pressing priority.

1. Advancing personalization in CTR

One of the most promising research frontiers lies in the personalization of CTR interventions using AI and machine learning (ML) [66,67]. AI-driven adaptive training models could dynamically adjust rehabilitation programs based on real-time patient feedback, optimizing exercise intensity, dietary adjustments (if necessary), and mental health interventions based on an individual’s physiological response, engagement level, and risk profile. Future research should explore how predictive analytics can anticipate rehabilitation plateaus, prevent early dropout, and enhance long-term adherence to CR. Also, ethical considerations surrounding AI-driven CTR are of a great interest in order to ensure their safe and effective clinical implementation.

2. Addressing accessibility and health disparities

Despite the scalability of CTR, health disparities remain a major barrier. Many populations, especially those in low-income, rural, or digitally underserved regions, lack the necessary technological infrastructure, digital literacy, or financial resources to fully engage in telerehabilitation. Thus, future research must focus on developing hybrid CTR models that blend home-based digital rehabilitation with periodic in-person support to increase accessibility, exploring the feasibility of low-cost, offline-compatible mHealth solutions and also investigating sex- and age-specific barriers to CTR, particularly among older adults and women. The integration of AI in CTR may help bridge some of these disparities by providing automated triage and tailored rehabilitation plans, potentially improving outcomes in underserved communities [67].

3. Evaluating long-term outcomes

Most current CTR studies primarily assess short-term physiological metrics like VO_2_ max improvements, blood pressure reductions, and lipid profile changes. However, long-term clinical and behavioral outcomes remain poorly characterized. Future research should shift toward “real-world” effectiveness studies examining CTR’s impact on MACE, hospital readmissions and long-term mortality, the influence of behavioral and psychological factors (patient motivation, depression, anxiety, social support networks), and how CTR influences long-term healthcare utilization and its cost-effectiveness compared to traditional, center-based rehabilitation.

4. Integrating emerging technologies in CTR

The rapid expansion of DHIs offers novel avenues for CTR optimization. Future studies should explore wearable biosensors and remote monitoring devices to enhance real-time physiological tracking and early risk detection, virtual reality (VR), and gamified rehabilitation to improve patient engagement and adherence to exercise regimens, and blockchain technology for secure health data management, ensuring privacy, interoperability, and seamless integration of CTR with electronic health records (EHRs).

5. Redefining the future of CR through CTR

The future of CR lies in developing equitable, scalable, and clinically validated CTR models that seamlessly integrate into broader healthcare ecosystems. This requires refining methodologies, enhancing personalization, and addressing accessibility gaps. A multidisciplinary research approach, involving cardiology, digital health, behavioral science, and health economics, is essential to establish CTR as a standard, evidence-based component of secondary prevention strategies worldwide.

Addressing these gaps is both a scientific necessity and a critical public health priority. Looking ahead, the future of CTR is poised for significant advancements driven by emerging technologies and evolving healthcare models. Regulatory frameworks and healthcare policies will also play an important role in shaping the scalability and accessibility of CTR, making it a cornerstone of CR in the near future.

## 4. Conclusions

This narrative review included fifteen studies, along with three additional studies—a retrospective, cross-sectional, and observational study—that demonstrated the potential for using smart devices as screening tools in CTR. The results across these studies emphasize the feasibility, safety, and efficiency of CTR interventions, with consistent improvements in CVRF, physical fitness, and patient satisfaction. In particular, the integration of smart technology and virtual consultations has proven to be a viable approach for monitoring and managing patients, particularly those at low risk following an acute myocardial infarction (AMI).

The selected and analyzed studies illustrate the potential for wearable devices and mobile applications to enhance the delivery of CR programs, with positive impacts on physical activity, cardiovascular health, and QoL. High adherence rates, patient satisfaction, and measurable improvements in VO_2_ max, exercise tolerance, and CVRF are key indicators of the efficacy of these interventions. However, some findings, such as the lack of significant differences in MACE and blood pressure profile changes in certain studies, highlight areas where further refinement and investigation are needed.

In conclusion, while the reviewed studies affirm the potential of CTR, addressing the identified limitations and gaps is essential for expanding its reach and impact. As digital health technologies evolve, incorporating AI-driven personalization, real-time monitoring, and hybrid CR models will further enhance CTR’s effectiveness. With further research and investment, CTR programs can become a cornerstone of post-AMI care, improving patient outcomes, reducing healthcare inequities, and enhancing long-term cardiovascular health.

## Figures and Tables

**Table 1:** The inclusion and exclusion criteria employed for selecting the studies for the narrative review.

Type	Inclusion Criteria	Exclusion Criteria
Type of Article	Randomized Controlled Trials, Prospective/Retrospective Cohort Study, Original Study, Pilot Study, Open Access publications	Narrative Review, Critical Review, Closed Access publications (full-text only available for a fee)
Population	Male and female patients following a myocardial infarction (STEMI) or acute coronary syndrome (Non-STEMI, unstable angina)	Cardiac patients without a history of myocardial infarction or acute coronary syndrome, including those with heart failure with reduced ejection fraction (HFrEF) or preserved ejection fraction (HFpEF), hypertension, post-transcatheter aortic valve implantation (TAVI), arrhythmias, chronic coronary syndrome, bypass grafting (CABG), significant neurological and psychological disorders, mobility impairments, and the very elderly
Interventions	Telemedicine or cardiac telerehabilitation involving home-based patients, smart wearable devices, exercise programs, and dietary interventions	Cardiac rehabilitation programs conducted in hospitals without a home-based component
Outcomes	Patient compliance with treatments, level of improvement in heart function after telerehabilitation, percentage of patients who experienced or did not experience a new heart attack, accessibility to smart devices, efficiency of online communication, risk/benefit analysis, and the percentage of patients from rural and urban environments	Articles lacking a significant impact on the role of post-infarction cardiac telerehabilitation or those that did not monitor cardiovascular risk factors

## Data Availability

Not applicable.

## Abbreviations

The following abbreviations are used in this manuscript:

AI	Artificial Intelligence
AMI	Acute myocardial infarction
AHA/ACC	American Heart Association/American College of Cardiology
BMI	Body mass index
CABG	Coronary artery bypass graft
CR	Cardiac rehabilitation
CTR	Cardiac telerehabilitation
CVD	Cardiovascular disease
CVRF	Cardiovascular risk factor
DHI	Digital health interventions
eHealth	Electronic health
EHRs	Electronic health records
ESC	European Society of Cardiology
GDPR	General Data Protection Regulation
HDL-C	High-density lipoprotein cholesterol
HFpEF	Heart failure with preserved ejection fraction
HFrEF	Heart failure with reduced ejection fraction
iCBT	Internet-based cognitive behavioral therapy
ICER	Incremental Cost-Effectiveness Ratio
IMDRF	International Medical Device Regulators Forum
IoT	Internet of Things
MACE	Major adverse cardiovascular events
METs	Metabolic equivalents
mHealth	Mobile health
ML	Machine Learning
PCI	Percutaneous coronary intervention
QALY	Quality-Adjusted Life Years
RCT	Randomized controlled trial
ROI	Return on investment
SaMD	Software as a medical device
SBP	Systolic blood pressure
STEMI	ST-elevation myocardial infarction
TAVI	Transcatheter aortic valve implantation
VO_2_ max	Maximum rate of oxygen consumption
VR	Virtual reality
VV	Virtual visit
